# Rethinking AI in youth mental health: promise, perils, and ethical integration

**DOI:** 10.1186/s13033-026-00714-z

**Published:** 2026-06-29

**Authors:** Andy Man Yeung Tai, Ian Hickie, Will Capon, Ante Prodan, Agam Sanghera, Michael Krausz, Frank Iorfino

**Affiliations:** 1 Department of Psychiatry, Faculty of Medicine, University of British Columbia, Vancouver, BC Canada; 2 Brain and Mind Centre, The University of Sydney, Sydney, Australia; 3 Program Neuroscience Postdoctoral Teaching and Learning Fellow, Department of Statistics ESB 3161, University of Toronto, University of British Columbia, 2207 Main Mall, Vancouver, BC V6T 1Z4 Canada; 4 Faculty of Science, Department of Statistics, The University of British Columbia, Vancouver Campus, Musqueam Traditional Territory, Vancouver, BC Canada

## Abstract

The transformative role of Large Language Models (LLMs) in youth mental health care as intelligent agents that can support patient engagement and augment healthcare delivery has been explored in this commentary. By acting as personalized virtual assistants for both patients and providers, LLMs have the potential to offer real-time, context-aware support, improving administrative workflows within mental health services. However, there is increasing concern within the research community regarding overinflation of claims surrounding large language models in mental healthcare. Technology companies often overstate their capabilities, creating unrealistic expectations that may lead to detrimental outcomes for patients. This paper critically examines the opportunities and limitations, advocating for greater transparency and rigorous scrutiny in the application of AI within sensitive domains such as mental health. It underscores the need for developmentally appropriate engagement, cultural sensitivity, and the integration of human expertise alongside AI to ensure ethical and effective collaboration in mental healthcare interventions.

## Introduction

Generative AI models, including ChatGPT, Llama, Bard, DeepSeek, and Claude, have recently emerged as key areas of research due to their potential applications as conversational agents in mental health [1]. These Large Language Models (LLMs) are highly regarded for their ability to perform multi-task analyses, deliver educational content, facilitate diagnoses, and support care management. The increasing adoption of AI within healthcare has raised expectations regarding the role of these technologies in enhancing mental health services and improving patient outcomes. However, despite these promising applications, the rapid proliferation of LLMs in healthcare has been accompanied by exaggerated claims from technology companies, often resulting in misconceptions about their actual capabilities [2, 3].

This paper seeks to examine the present and potential applicability of LLMs in the context of mental health, with particular emphasis on the complex challenges faced by young people who require personalized, developmentally appropriate care. The unique neurocognitive stages of adolescence, coupled with specific social dynamics such as peer influence and identity formation, require interventions that are highly tailored. While LLMs offer potential efficiencies, such as automating administrative tasks or augmenting documentation processes, currently these models are in their infancy in the realm of youth mental health. Consequently, they frequently lack the deep understanding necessary for accurately assessing the emotional needs of this demographic [4].

Moreover, the pervasive marketing of LLMs as revolutionary tools in mental healthcare raises concerns about the disconnect between the purported capabilities of these systems and their practical performance. This leads to inflated expectations in clinical and policy contexts. Mental health care for younger demographics poses distinctive challenges that current AI systems are only beginning to address. The developmental complexity requires an empathetic, tailored approach, and the therapeutic alliance between clinician and patient remains essential for effective care. AI systems can provide useful adjunctive support and aid human expertise, which is critical for guiding treatment decisions when working with vulnerable populations.

Integrating LLMs into mental health care presents important considerations that can enhance their effectiveness when addressed thoughtfully. AI models must be trained on diverse and representative datasets to ensure equitable care, particularly for individuals from marginalized or underserved communities. While models like ChatGPT have been trained on an extensive range of internet data with minimal filtering, proactive measures such as refining training processes and implementing culturally sensitive frameworks can mitigate potential biases [4]. Additionally, bridging the digital divide by expanding access to AI-driven solutions in rural and low-income communities can help ensure that technological advancements benefit all individuals equitably.

## Operational efficiency

Large Language Models have the potential to significantly enhance operational efficiency within clinical settings, particularly in mental health services where heavy caseloads and limited staffing often strain resources. These AI tools can automate a range of administrative tasks, such as transcribing therapy sessions, summarizing patient notes, scheduling appointments, and managing billing processes. By streamlining these labor-intensive activities, LLMs enable clinicians to focus more on direct patient care and early intervention efforts [5].

The limiting factor for most LLMs is the computational and monetary cost entailed in training. OpenAI’s GPT models contain over 100 billion trainable parameters. These large networks require substantial investment in hardware, time, and energy. There has been some development to combat this limitation recently. Recent advancements highlight the rapid evolution of LLM development and its increasing accessibility. DeepSeek AI has demonstrated that developing a high-performing LLM does not necessarily require hundreds of millions of dollars and extensive development timelines. Reports suggest that DeepSeek AI achieves comparable performance to OpenAI’s GPT-4 while operating at a fraction of the computational cost [17], potentially reducing barriers to model development in clinical applications. This shift lowers the barrier for the creation of specialized LLMs tailored to mental health services, suggesting that AI-driven solutions may soon become more feasible for resource-constrained settings.

Though training large-scale models can be expensive, several technical strategies can reduce costs while maintaining performance. Model compression techniques (e.g., quantization, pruning, or knowledge distillation) reduce the memory footprint and energy consumption of LLMs. This makes it easier for smaller clinics to deploy AI solutions on-premises or via cost-effective cloud services.

New tools that work in tandem with mental health services in Australia demonstrate how AI can assist clinicians by providing real-time tracking of mental health trajectories [6]. Such tools can flag significant changes in a patient’s behavior or outcomes, allowing clinicans to prioritize cases requiring urgent attention. Rather than replacing human oversight, these systems complement clinical judgment to support the timely delivery of care. Additionally, LLMs offer operational improvements in areas like billing and insurance processing. Systems such as Epic In-basket and Waystar leverage AI to facilitate the electronic submission of claims and categorize patient information [7, 8]. However, it is critical to recognize that LLMs should be viewed strictly as supportive administrative tools in these contexts.

### Clinical decision-making and human-AI collaboration

The integration of AI tools into clinical workflows offers numerous advantages that can improve patient outcomes. AI can process vast amounts of data quickly, providing clinicians with valuable insights by identifying patterns or potential red flags that might be difficult for humans to detect in real time. This ability to rapidly analyze data can be particularly beneficial in high-demand environments where timely intervention is crucial [9]. By working in tandem with AI, clinicians can enhance their decision-making processes. LLMs can assist by generating evidence-based suggestions, offering clinical guidelines, and providing reminders of best practices [10].

In high-demand mental health environments, AI tools can also go beyond traditional diagnostic support by offering real-time triage and risk management. LLMs that monitor symptom logs or intake forms can be configured to detect subtle language patterns indicative of escalating anxiety or depressive symptoms. When such patterns emerge, the system can flag these cases for immediate clinical attention. This approach reduces the likelihood of acute crises and ensures that limited mental health resources are directed to those who most urgently need care.

For comprehensive care, decisions often require input from psychologists, social workers, educational counselors, and other professionals. LLMs can serve as a centralized platform that aggregates diverse data streams, from school performance reports to peer feedback, to generate a holistic clinical snapshot. By presenting a unified view of the patient’s circumstances, these systems encourage meaningful collaboration. This fosters a coordinated approach where each professional’s insights are integrated with AI-driven summaries, resulting in an informed consensus on treatment plans tailored to a patient’s developmental and social context.

Developing culturally sensitive AI models presents significant challenges in this domain. The primary obstacle is the severe lack of diverse, developmentally appropriate clinical training data, which often results in models biased toward adult or Western populations. Potential enablers to overcome these challenges include community-participatory AI design, which ensures diverse demographic representation during the model development phase. Furthermore, the adoption of privacy-preserving machine learning techniques, such as federated learning, allows for the training of models across distributed clinical sites without centralizing sensitive patient data. This approach facilitates the construction of more representative and culturally aware models while strictly maintaining patient confidentiality.

A key concern in collaborative clinical workflows is ensuring clinicians understand why an AI system has generated a particular recommendation. Techniques such as attention visualization or feature-importance scoring allow mental health professionals to see the underlying rationale behind AI-derived suggestions. By providing transparent explanations, LLMs empower clinicians to assess whether the system’s reasoning aligns with known clinical guidelines. This added layer of interpretability helps maintain trust in AI-driven decision support.

### Warning: attention is not all you need

A significant concern is the overpromotion of LLM capabilities by technology companies, leading healthcare providers and patients to mistakenly believe that AI systems can replace human expertise. This is particularly problematic in developmentally appropriate mental health care, where misdiagnosis or inappropriate treatment can result in severe consequences. Non-experts may implement AI systems without fully understanding their limitations, leading to misallocated resources and suboptimal care. In the worst cases, LLMs could propagate misinformation and cause harm to vulnerable populations.

For major impact in this field, LLMs must be trained on data reflecting the unique developmental experiences of young people [11]. AI systems designed for broader populations may overlook how mental health symptoms manifest differently in adolescents. This longstanding issue in mental health research has led to a bias in our understanding of disorders, often favoring adult symptom presentations [12]. These presentations are typically highly comorbid and often the result of chronicity, which impacts the accurate understanding of the onset and course of disorder in youth. For example, youth may express subthreshold and mixed symptoms profiles, whereas in adulthood the syndromes tend to be more stable and distinct [13, 14]. Developmentally specific training data is therefore essential to ensure AI tools are relevant.

Despite the support LLMs provide in analyzing patient data, it is unclear to what extent they will be able to effectively imitate or transform the therapeutic relationship between clinician and patient. Trust and rapport are crucial, and AI tools should enhance, not compromise, the human element of care. Advanced technologies enable real-time interactions that enhance the delivery of digital care [15]. However, AI-driven empathy is limited. While they can simulate human emotions, their ability to provide effective emotional validation is a functional limitation. Over-reliance on AI risks weakening the essential therapeutic bond between clinician and patient. Human clinicians offer emotional intelligence and cultural understanding that AI cannot replicate. Additionally, LLMs often struggle with culturally specific expressions [16]. AI tools must complement, not replace, the clinical judgment required to deliver effective, personalized care.

## Conclusion: proceeding with caution

As we move forward, it is crucial for the mental health community to both embrace the opportunities of AI and temper expectations about LLMs with transparency and ethical awareness. While AI-driven tools can complement clinical care, there is a clear need for human clinicians who understand each patient’s unique developmental and emotional context. AI should support human judgment, ensuring that interventions are personalized and aligned with each patient’s specific needs.

Though LLMs offer significant potential for enhancing efficiency, there are inherent risks if their capabilities are overstated. Clear ethical guidelines and regulatory oversight are essential to prevent AI from undermining clinical practices. In such a rapidly developing area, it is important that these approaches remain flexible to support safe innovation. AI must be viewed as a collaborative tool that enhances care without compromising emotional intelligence, cultural sensitivity, or clinical judgment. By setting realistic expectations and maintaining human oversight, we can ensure that patients receive the empathetic, personalized care they deserve in a rapidly evolving healthcare landscape.

## Data Availability

No datasets were generated or analysed during the current study.

## References

[1] Cheng SW, Chang CW, Chang WJ, Wang HW, Liang CS, Kishimoto T, et al. The now and future of ChatGPT and GPT in psychiatry. Psychiatry Clin Neurosci. 2023;77(11):592–6. 37612880 10.1111/pcn.13588 PMC10952959

[2] Thirunavukarasu AJ, Ting DSJ, Elangovan K, Gutierrez L, Tan TF, Ting DSW. Large language models in medicine. Nat Med. 2023;29(8):1930–40. 37460753 10.1038/s41591-023-02448-8

[3] Greene TTNW, Deep-Tech. 2022 [cited 2024 Oct 16]. Meta takes new AI system offline because Twitter users are mean. Available from: https://thenextweb.com/news/meta-takes-new-ai-system-offline-because-twitter-users-mean

[4] Harrer S. Attention is not all you need: the complicated case of ethically using large language models in healthcare and medicine. eBioMedicine. 2023;90. Available from: https://www.thelancet.com/journals/ebiom/article/PIIS2352-3964(23)00077-4/fulltext 10.1016/j.ebiom.2023.104512 PMC10025985 36924620

[5] Blease C, Worthen A, Torous J. Psychiatrists’ experiences and opinions of generative artificial intelligence in mental healthcare: An online mixed methods survey. Psychiatry Res. 2024;333:115724. 38244285 10.1016/j.psychres.2024.115724

[6] Varidel M, Hickie IB, Prodan A, Skinner A, Marchant R, Cripps S, et al. Dynamic learning of individual-level suicidal ideation trajectories to enhance mental health care. Npj Ment Health Res. 2024;3(1):1–9. 38849429 10.1038/s44184-024-00071-0 PMC11161660

[7] JMIR Research Protocols. Validation of the InnoWell Platform: Protocol for a Clinical Trial. Available from: https://www.researchprotocols.org/2019/5/e13955 10.2196/13955 PMC6658233 31152524

[8] Epic EHR integration, editor. Waystar. Available from: https://www.waystar.com/epic-integration/

[9] O’Sullivan JW, Palepu A, Saab K, Weng WH, Cheng Y, Chu E et al. Towards Democratization of Subspeciality Medical Expertise. arXiv; 2024. Available from: http://arxiv.org/abs/2410.03741

[10] Tu T, Palepu A, Schaekermann M, Saab K, Freyberg J, Tanno R et al. Towards Conversational Diagnostic AI. arXiv; 2024. Available from: http://arxiv.org/abs/2401.05654

[11] Xu X, Yao B, Dong Y, Gabriel S, Yu H, Hendler J, et al. Proc ACM Interact Mob Wearable Ubiquitous Technol. 2024;8(1):31:1–3132. Mental-LLM: Leveraging Large Language Models for Mental Health Prediction via Online Text Data. 10.1145/3643540 PMC11806945 39925940

[12] Right. care, first time: a highly personalised and measurement based care model to manage youth mental health. Hickie, 2019. Medical Journal of Australia. Available from: https://onlinelibrary.wiley.com/doi/full/10.5694/mja2.50383 10.5694/mja2.50383 31679171

[13] Scott J, Iorfino F, Capon W, Crouse J, Nelson B, Chanen AM, et al. Staging 2.0: refining transdiagnostic clinical staging frameworks to enhance reliability and utility for youth mental health. Lancet Psychiatry. 2024;11(6):461–71. 38643773 10.1016/S2215-0366(24)00060-9

[14] Iorfino F, Scott EM, Carpenter JS, Cross SP, Hermens DF, Killedar M, et al. Clinical Stage Transitions in Persons Aged 12 to 25 Years Presenting to Early Intervention Mental Health Services With Anxiety, Mood, and Psychotic Disorders. JAMA Psychiatry. 2019;76(11):1167–75. 31461129 10.1001/jamapsychiatry.2019.2360 PMC6714017

[15] Hippocratic AI, Announces Collaboration with NVIDIA. Available from: https://www.globenewswire.com/news-release/2024/03/18/2848236/0/en/Hippocratic-AI-Announces-Collaboration-with-NVIDIA-to-Develop-Super-Low-Latency-Empathy-Inference-for-One-of-the-World-s-First-Generative-AI-Powered-Healthcare-Agents.html

[16] Cross-Cultural Implications of Large Language Models. An Extended Comparative Analysis. SpringerLink. Available from: https://link.springer.com/chapter/10.1007/978-3-031-76806-4_8

[17] DeepSeek-AI. DeepSeek-V3 Technical Report. arXiv. 2024. Available from: https://arxiv.org/abs/2412.19437

